# Challenges in Medicine, Magnified by the Pandemic: A Dual Battle for Female Physicians

**DOI:** 10.7759/cureus.62354

**Published:** 2024-06-14

**Authors:** Huma Farid, Amy Sullivan, Ajayi Ayodele, Annliz Macharia, Katharyn M Atkins

**Affiliations:** 1 Obstetrics and Gynecology, Beth Israel Deaconess Medical Center, Harvard Medical School, Boston, USA; 2 Shapiro Center for Medical Education, Beth Israel Deaconess Medical Center, Harvard Medical School, Boston, USA

**Keywords:** healthcare workplace culture, initial retention force, physician wellbeing, work-life balance, world pandemic

## Abstract

Introduction: We aimed to understand how the pandemic impacted work hours and employment status of female physicians.

Methods: An anonymous survey of female physicians was distributed through social media and email lists from 12/2021 to 2/2022. Primary outcomes were changes in physicians’ work schedules and employment status. Analyses included descriptive statistics of closed-ended items and qualitative content analysis of open-ended responses.

Results: We restricted our analysis to four specialties: obstetrics and gynecology, internal medicine, anesthesia, and pediatrics (n=626). The majority (92%) of respondents had caretaking responsibilities; 43% changed work schedules to accommodate those responsibilities. Around 17% of physicians changed jobs. The most common reasons for job changes included: negative work environment, lack of work-life balance, burden of work, and lack of efforts to mitigate COVID-19.

Conclusion: The pandemic highlighted the need for flexibility, improvements in workplace culture, and financial incentives to increase retention.

## Introduction

Dr. Elizabeth Blackwell, the first female physician in America, wrote, “A blank wall of social and professional antagonism faces the woman physician that forms a situation of singular and painful loneliness, leaving her without support, respect or professional counsel” [1]. Although Dr. Blackwell died over a century ago, her words still carry a remnant of truth. Female physicians remain burdened by dual societal and professional expectations and face challenges both at home and at work. 

Women have historically borne the brunt of childcare and household management tasks [2], and women in medicine are no different in being subject to these societal expectations. Prior to the pandemic, in dual-physician households, female physicians with children and male partners spent an average of 100 minutes more per day on childcare and household tasks than their partners, even after adjusting for work hours [3].

Gender discrepancy persists at work as well, with physician mothers reporting limited opportunities for career growth, unequal pay compared to male colleagues, limited or nonexistent support from employers around pregnancy and lactation, lack of adequate childcare, and the encroachment of work-related activities on time at home [4]. Female physicians noted a dearth of role models who had successfully balanced career and family responsibilities [5]. A cross-sectional survey of female physicians found that 66% had experienced gender discrimination, and 36% had experienced discrimination specifically because of becoming a mother [6]. Nearly half of female physicians surveyed in the Women Physicians’ Health Study reported experiencing gender-based harassment [7]. Burnout rates are higher for women physicians than their male counterparts [8].

During the pandemic, an even greater proportion of the burden likely fell on women physicians’ shoulders. For example, among female physicians with preschool-aged children, work hours and income decreased [9], likely due to lack of childcare. School and daycare closures, as well as disruptions in childcare due to exposure to or infection with COVID-19, led to gaps in reliable, consistent childcare; simultaneously, physicians were required to work longer hours during each subsequent wave of infection [10]. In one study of 6,466 healthcare workers, 23% of female healthcare workers reported exhaustion due to caregiving responsibilities, both at home and at work [11]. In addition, female physicians with children were more likely to report burnout [12]. Another study of 276 physician mothers demonstrated that women physicians shouldered more responsibilities related to childcare and household tasks during the pandemic, experienced more conflict between dueling responsibilities of work and home, and had a higher risk of depression than male physicians with children [13]. 

The ripple effects of the pandemic continue to be felt; even after the first wave of the pandemic, women physicians in 2021 reported increasing amounts of work-related stress [14]. The primary objective of this study was to understand how the pandemic impacted work schedules and employment status of female physicians during the initial two years of the pandemic. The secondary objective was to understand whether this differed among female physicians with and without caretaking responsibilities. 

## Materials and methods

We conducted an observational cohort study of female physicians in the United States (US) from 12/2021 to 2/2022 using an anonymous survey. The survey was distributed nationally through email listserves. It was also posted online on popular social media outlets with a broad national reach, such as the Physician Mom Group on Facebook, a national group with about 80,000 female physician members. The only inclusion criterion was that participants identified as female physicians; they did not need to be currently practicing, but needed to have completed medical school. Although physicians from multiple specialties responded, the analysis was limited to physicians in anesthesia, internal medicine, obstetrics and gynecology, and pediatrics, as these groups had the most respondents. Sample calculation was not performed.

The survey specifically focused on the experiences of female physicians during the pandemic and was developed through pilot testing with four physicians and an expert in survey design. The survey was discussed with each person who piloted it to refine survey questions. (The survey can be found in Appendix A). Of note, the survey questions examining feelings of burnout were adapted from the Maslach burnout inventory [15]. The types of questions in the questionnaire (e.g. physicians' employment status, work, and personal schedules, caretaking responsibilities, thoughts of quitting) were included to better elicit a thorough picture of each respondent's experiences as a female physician during the pandemic. 

Data are presented as frequency (percent). The primary outcomes of interest (changes in physicians' work schedules and reasons for changes in employment status) were stratified by specialty. All analyses were performed using SAS 9.4 (SAS Institute Inc., Cary, NC, USA). Open-ended survey responses were reviewed independently by three investigators (HF, KMA, AS), read in their entirety, and then coded using a content analysis approach and inductive coding. A codebook was created through an iterative process; team members met multiple times to revise the codebook while discussing open-ended survey responses. Coding discrepancies were resolved through discussion, and a final code was assigned to each open-ended response after unanimous agreement. We used descriptive statistics to analyze the study sample and featured representative quotations that reflected common themes. Our institutional review board approved this study (Protocol Number 2021P000573).

## Results

A total of 1169 participants completed the survey, but only a subset of those participants (626 physicians or 53.5%) were included in the final analysis as they worked in one of the four most common specialties. In total, 303 (48%) were obstetrician-gynecologists; 146 (23%) were internal medicine, 96 (15%) were anesthesiologists, and 81 (13%) were pediatricians.

A substantial proportion of respondents (222, 35%) actually increased their work hours during the pandemic. Only 53 (8%) of respondents changed to part-time status or became unemployed, ranging from 17 (6%) among obstetrician-gynecologists to 14 (15%) among anesthesiologists. Even if they didn’t quit their jobs, many physicians thought about it, with 405 (65%) of physicians thinking about quitting their jobs monthly or weekly, and 85 (14%) of respondents thinking about quitting daily (Table 1).

**Table 1:** Characteristics of 626 female physician respondents, stratified by specialty

Characteristics	All Respondents n=626 (%)	Anesthesia n=96 (%)	Internal Medicine n=146 (%)	Obstetrics and Gynecology n=303 (%)	Pediatrics n=81 (%)
Age (years)					
<40	252 (40)	37 (39)	62 (42)	127 (42)	26 (32)
40-49	290 (46)	47 (49)	64 (44)	138 (46)	41 (51)
≥50	83 (13)	12 (13)	20 (14)	38 (13)	13 (16)
Missing	1 (0.2)	0 (0)	0 (0)	0 (0)	1 (1)
Self-reported race and ethnicity					
Hispanic	19 (3)	3 (3)	4 (3)	12 (4)	0 (0)
Non-Hispanic Asian	80 (13)	11 (11)	29 (20)	28 (9)	12 (15)
Non-Hispanic Black	16 (3)	2 (2)	1 (1)	12 (4)	1 (1)
Non-Hispanic White	472 (75)	75 (78)	98 (67)	239 (79)	60 (74)
Another race/ethnicity	38 (6)	5 (5)	14 (10)	12 (4)	8 (10)
Married	570 (91)	84 (88)	131 (90)	277 (91)	78 (96)
Type of work					
Private practice	222 (35)	41 (43)	22 (15)	130 (43)	29 (36)
Academic institution	277 (44)	43 (45)	107 (73)	95 (31)	32 (40)
Community institution	118 (19)	9 (9)	15 (10)	74 (24)	20 (25)
Government institution	9 (1)	3 (3)	2 (1)	4 (1)	0 (0)
Currently in training	25 (4)	2 (2)	12 (8)	9 (3)	2 (2)
Region					
Northeast	291 (46)	30 (31)	106 (73)	106 (35)	49 (60)
Midwest	101 (16)	18 (19)	7 (5)	71 (23)	5 (6)
South	109 (17)	25 (26)	11 (8)	66 (22)	7 (9)
West	122 (19)	23 (24)	21 (14)	58 (19)	20 (25)
Missing	3 (0.5)	0 (0)	1 (1)	2 (1)	0 (0)
Employed full-time	513 (82)	78 (81)	108 (74)	280 (92)	47 (58)
Partner employed outside home	487 (78)	71 (74)	116 (79)	227 (75)	73 (90)
Changed work hours due to pandemic					
Decreased hours	102 (16)	30 (31)	18 (12)	38 (13)	16 (20)
Increased hours	222 (35)	31 (32)	78 (53)	89 (29)	24 (30)
No change	302 (48)	35 (36)	50 (34)	176 (58)	41 (50)
Changed employment status					
Changed to part-time/unemployed	53 (8)	14 (15)	13 (9)	17 (6)	9 (11)
Changed to full-time	7 (1)	0 (0)	4 (3)	3 (1)	0 (0)
No change in status	566 (90)	82 (85)	129 (88)	283 (93)	72 (89)
Frequency of thoughts of quitting					
Daily	85 (14)	10 (10)	24 (16)	34 (11)	17 (21)
Weekly	188 (30)	32 (33)	42 (29)	97 (32)	17 (21)
Monthly/few times per year	217 (35)	29 (30)	53 (36)	105 (35)	30 (37)
Never	135 (22)	25 (26)	27 (18)	67 (22)	16 (20)
Missing	1 (0.2)	0	0	0	1 (1)
Changed job	108 (17)	15 (16)	23 (16)	55 (18)	15 (19)
Had caretaking responsibilities	577 (92)	89 (93)	129 (88)	284 (94)	75 (93)
Childcare	553 (96)	88 (99)	119 (92)	274 (96)	72 (96)

A total of 108 (17%) physicians changed to another job in medicine during the pandemic (Table 1), and the proportions were similar across specialties: 23 (16%) internal medicine physicians, 15 (16%) anesthesiologists, 55% obstetrician-gynecologists, and 15 (19%) pediatricians. One anesthesiologist explained why she left her job: “We felt extremely undervalued and unsupported.” An internal medicine physician wrote, “I did not feel valued or appreciated. I didn’t have time to get to know my patients as people or learn their stories …The tasks and checkboxes and documentation was never-ending.” A pediatrician described an “extremely unsupportive environment, lack of resources, no acknowledgment of COVID-related stressors on employees and staff, poor culture in the organization” as reasons why she left her job.

**Figure 1:**
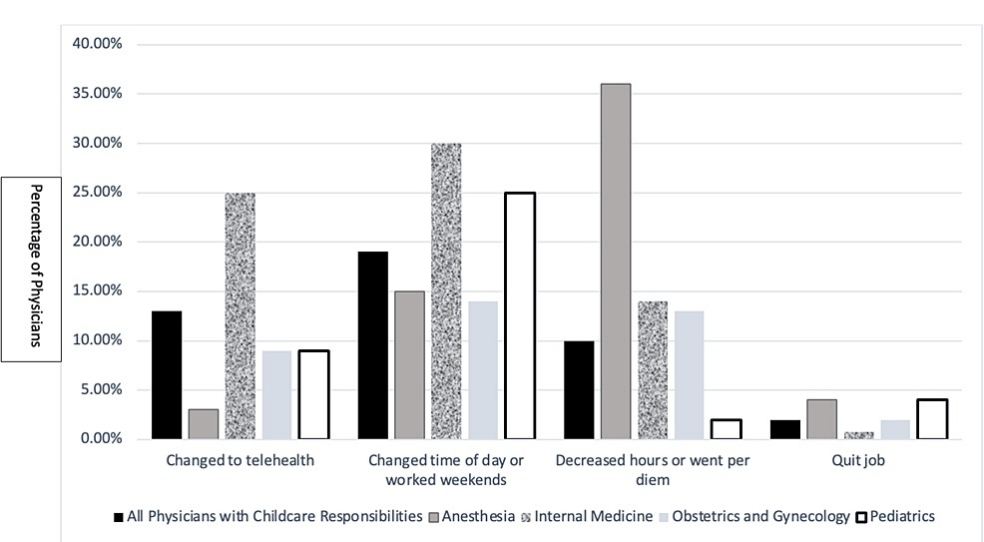
Changes in work schedule due to childcare responsibilities

We then examined physicians with caretaking responsibilities. The majority of respondents (577, or 92%) had caretaking responsibilities, with nearly all (553, or 96%) of caretaking responsibilities focused on caring for children (Table 1). A total of 246 (43%) female physicians with childcare responsibilities changed their work schedules due to those responsibilities. Of those respondents, 77 (13%) changed to working telehealth; 111 (19%) changed the time of day they worked or worked weekends; 60 (10%) decreased their hours or went to per diem; 12 (2%) quit their job (Figure 1). When examined by specialty, internal medicine physicians more frequently changed to working telehealth (41 respondents, or 32%) while anesthesiologists were the least likely to do so (3 respondents, or 3%). A total of 32 (25%) internal medicine respondents and 14 (19%) pediatricians were more likely to change the time of day they worked or work weekends compared to anesthesiologists or obstetricians-gynecologists. More anesthesiologists with caretaking responsibilities (14%) decreased hours or went to per diem compared to any other specialty (Figure 1).

**Figure 2:**
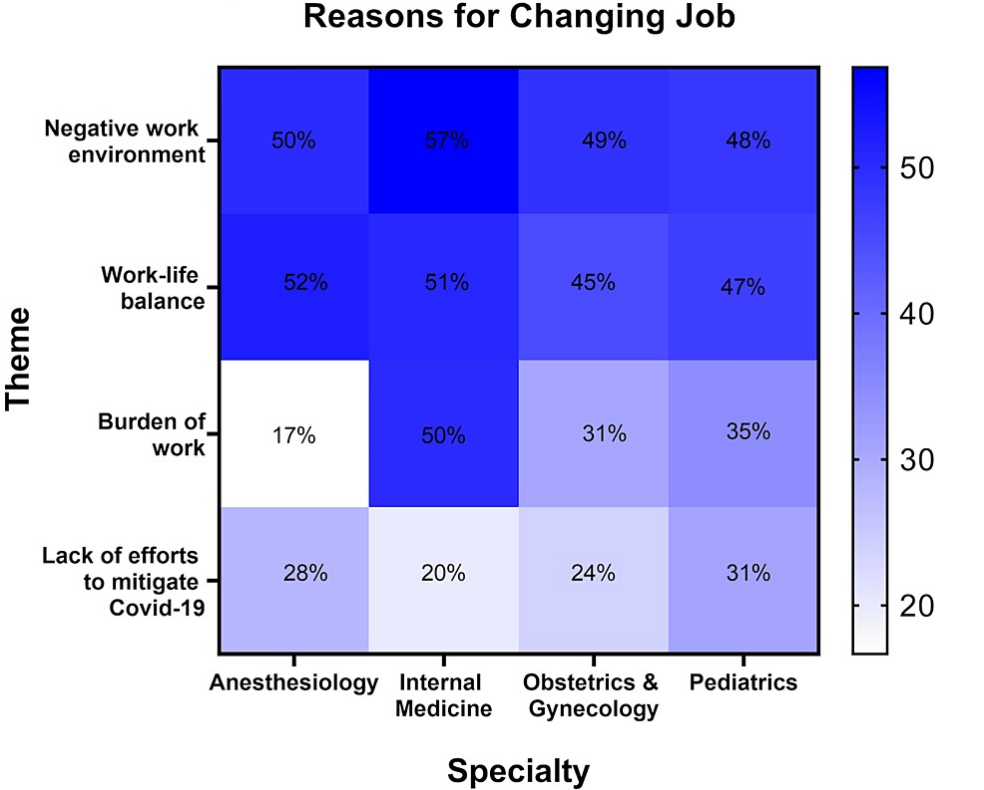
Reasons for changing jobs

We asked physicians why they changed to another job; analysis of the survey responses identified several major themes: negative working environment, lack of work-life balance, burden of work, and lack of efforts to mitigate COVID-19 (Figure 2).

An analysis of the open-ended survey results confirmed the themes found in Figure 2.

Negative work environment

Physicians reported negative work environments, describing patients who became angry with physicians who attempted to provide care in a “chaotic work environment.” A physician reported feeling “moral injury” at being unable to provide the kind of care she wanted. Physicians noted a lack of support from administrators or leadership and felt “expendable.” Respondents also commented on the lack of adequate compensation. They felt that they were working harder for less pay and that they were not provided with opportunities to advance their careers. Many physicians mentioned that hazard pay should have been considered for front-line physicians. 

Lack of work-life balance

Lack of work-life balance was a significant concern among physicians. Women described feeling conflicted as they tried to balance the demands of their jobs and their families. The distress that inadequate child care created was clear in the responses; physicians juggled their schedules in a variety of ways to accommodate inconsistent childcare. One obstetrician-gynecologist reported using her administrative days for childcare, and an internal medicine physician worked in the evenings and weekends to accommodate childcare. Another internal medicine physician had to “shorten clinic, cancel or make up outside of usual clinic hours due to preschool/daycare closure.” Multiple physicians noted the need for flexibility in schedules, with some being unable to go part-time due to financial demands, and others being denied the opportunity for flexibility. 

Burden of work

Physicians described feeling overwhelmed by the burden of work, with unsustainable workloads that “just kept increasing.” An internal medicine physician described being “completely exhausted” as she attempted to work on the COVID wards while meeting her family’s needs. This unsustainable workload led to significant burnout. One obstetrician-gynecologist reported an episode of severe depression during the pandemic that led to a suicide attempt. Another physician stated, “[Medicine] is not worth what it is costing me,” and yet another physician wrote, “I don’t love medicine anymore.” One pediatrician described, “I wish that our employer would acknowledge burnout in a meaningful way. I don't want to receive emails about meditation apps. I want them to fix the things that lead to burnout. I want them to provide appropriate PPE [Personal Protective Equipment]. I want them to listen carefully to feedback from people on the frontlines about patient care and service delivery. I want them to not cut our pay, and to compensate fairly for the extra work we are doing.” 

Lack of efforts to mitigate COVID-19

Amidst all of these concerns, physicians were being exposed to unsafe working conditions that did not mitigate the risk of COVID-19. Physicians worked in areas where there was significant anti-vaccine, anti-masking sentiment. Physicians reported a dearth of appropriate personal protective equipment and a lack of policies enforcing masking in the hospital or encouragement of vaccinations. A pregnant anesthesiologist recalled being asked to intubate patients infected with COVID-19 without an N95 mask. Another pregnant anesthesiologist was uncomfortable working in the operating room without routine COVID testing and inadequate PPE but was not offered alternative work options. 

Table 2 summarizes common themes and representative comments.

**Table 2:** “I don’t love medicine anymore:" themes identified through iterative coding

Theme	Excerpt
Negative work environment and lack of adequate compensation	“Chaotic work environment. Angry patients.” “I felt moral injury over not being able to provide care in a way that I wanted.” “Essentially there was zero support from administrators or leadership, and a complete lack of respect (snide comments, condescension) whenever unsafe working conditions were repeatedly brought up by female department members.” “They…treat us as expendable.” “Working harder for less and unable to meet financial responsibilities.” “[They should have] Provided appropriate and market rate compensation, valued me and my abilities, provided women with equal opportunity.” “Hazard pay may have helped.”
Lack of work-life balance and flexibility	“Hard to do it all—take care of little kids and work all the time.” “Feel my job keeps me from doing everything I need to do for my family.” “ My children are suffering for my lack of presence.” Inadequate childcare “Hard to do right by my patients while kids getting kicked out of daycare/preschool at such a low threshold.” “Super stressed about unreliable childcare and paying for childcare.” “Just gave my notice as [I am] unable to coordinate child care with pandemic and current work environment not supportive.” “[I] Would have liked to change hours but this could not be accommodated.” “Need for more flexible job hours due to childcare issues.”
Burden of work and burnout	“I feel like I can't take a break and they keep piling on more work.” “I felt unappreciated for the extra work that I was asked to do without any compensation or acknowledgment.” “I was burned out, became acutely depressed and attempted suicide.” “This is not worth what it is costing me.” “I don’t love medicine anymore.”
Lack of efforts to mitigate COVID-19	“Pregnant and employer wanted me to intubate Covid patients without allowing me n95.” “I was pregnant in March 2020 (due in July) and removed myself from the OR (I’m an anesthesiologist) due to inadequate COVID screening for surgical patients, inadequate PPE supplies, and unknown risks of COVID to pregnant mothers & fetuses…I …was denied any alternative to clinical OR work.” “Live in an area with high numbers of anti-science, anti-mask, anti-vax sentiments. It’s exhausting taking care of this patient population. Feels like I care more about their health than they do.”

Incentives for retention 

**Figure 3:**
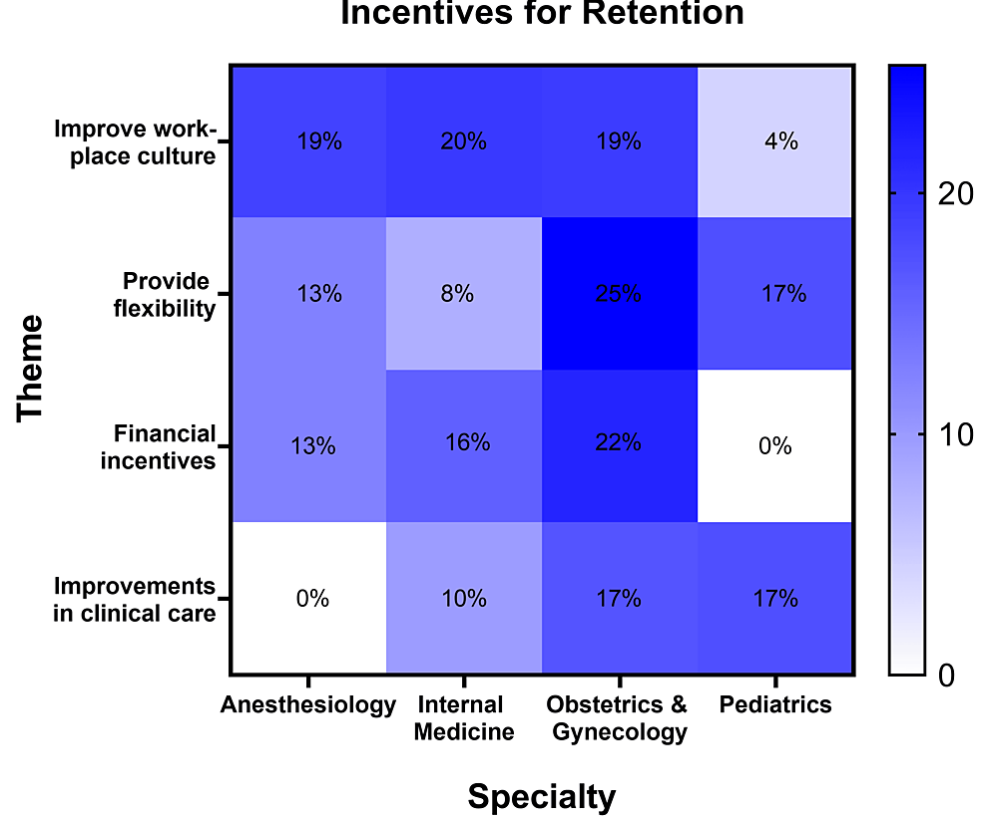
Incentives for retention

When physician respondents were asked what employers and institutions could do to improve female physician retention, the suggestions fell into several categories: improve workplace culture, provide flexibility, offer financial incentives, and improve clinical care (Figure 3). Physicians suggested hazard pay and increasing salary. Other requests were simple, such as for the employer to provide adequate personal protective equipment and offer the option of working from home and job-sharing opportunities. Physicians asked for decreases in administrative burden and providing coverage on days off, as well as increasing support staff and decreasing workload. One physician admonished her institution to “take the money you spent on the “heroes work here” signs and actually do something useful with it.” Respondents needed free mental health services and childcare backup, and they desired acknowledgment and recognition of their sacrifices and hard work as well as transparency among leadership. An anesthesiologist asked her leadership “to simply get on the front line with us,” and another anesthesiologist recommended “higher salary, fewer hours of work, more admin time and support, support with part-time or flexible work, support for childcare or other life responsibilities” as ways to increase retention.

## Discussion

In this cross-sectional survey of female physicians, the vast majority (92%) of whom had caretaking responsibilities, we analyzed changes in work schedules during the pandemic. We found that among those with childcare responsibilities, nearly half (43%) modified their work schedules due to those responsibilities. This included incorporating telehealth into their practice, shifting their work hours, or switching to working part-time, presumably to fill in care gaps secondary to school closures and disruptions in childcare. Not surprisingly, anesthesiologists were unable to incorporate telehealth or change their schedules when compared with other physicians, likely due to the exigencies of operating room schedules, but were more likely to become per diem as a direct response to challenges with childcare. 

**Figure 4:**
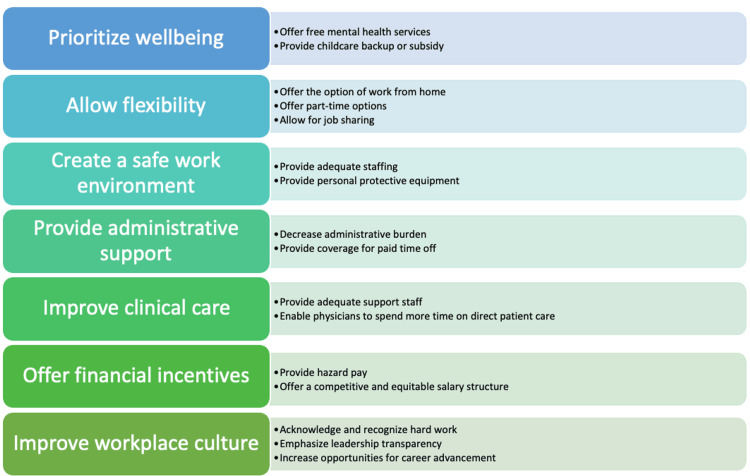
Guide to female physician retention

When evaluating systemic issues that impact physician job retention, we examined both the reasons why people quit their jobs and what they suggested institutions could do to promote retention. This analysis revealed that well-being could be seen as the crux of physician concerns, with several opportunities and areas for improvement. The figure below illustrates changes that institutions can implement to retain female physicians (Figure 4).

Wellbeing spans a broad spectrum of human needs; Maslow whittled these needs into five categories: physiological, safety and security, love and belonging, self-esteem, and self-actualization [16]. Our data revealed that simply focusing on wellbeing as acknowledgment through signs touting that “heroes work here” or giving gifts of gratitude was insufficient because it did not comprehensively or meaningfully address the full spectrum of wellbeing. Rather, reflection on the experiences of female physicians’ day-to-day lives allows us to understand the components of well-being, both at home and at work, that can improve physician retention. 

Negative work environments impact well-being. Our respondents described work environments that felt “chaotic” and created a sense of “moral injury.” Unsustainable, overwhelming workloads robbed physicians of the joy they experienced previously in medicine, some of whom developed depression, burnout, and other mental health issues. Working in hospitals that did not have adequate personal protective equipment or enforce masking or vaccinations made physicians feel unsafe and heightened their concerns about their health. The physicians who described these situations responded by quitting their jobs. 

On the home front, adequate, lack of reliable childcare remains a significant concern for women physicians, as demonstrated by our study and the work of Meese [11]. Many of the respondents described the tension created by trying to work while managing daycare and school closures throughout the pandemic. 

Prioritizing well-being requires examining all the facets that comprise women physician wellbeing and addressing each of those. Offering free mental health services could help to alleviate mental health issues and burnout. Creating a safe work environment with adequate staffing and personal protective equipment, diminishing the administrative burden on physicians, and providing adequate support staff would allow physicians to safely take care of their patients in a supportive workplace that would lead to improved clinical care. Providing backup childcare or a childcare subsidy and increasing options for flexibility, such as offering the option of working from home and allowing for job sharing and part-time options, could also improve retention and well-being. Financial incentives and a competitive and equitable salary structure would contribute to improvements in work-life balance and well-being as well. Ultimately, these initiatives would create a positive, transparent workplace culture where physicians are acknowledged for their work and receive opportunities for career advancement. 

The issues that female physicians face predated the pandemic. A literature review conducted prior to the pandemic demonstrated that female physicians’ needs include better work-life integration and flexible work hours [17]. However, the pandemic appears to have pushed these challenges to a critical point. Lack of work-life integration and the phenomenon of “double-duty caregiving,” i.e., of being a primary caregiver both at home and at work, has led to burnout [11]. During the pandemic, nearly 25% of female physicians were the primary caregivers and household managers compared to male physicians. Among dual physician households, physician mothers were 30 times more likely to take primary responsibility for their children’s schooling than physician fathers [13]; even more sobering, none of the physician fathers were primary caretakers for their children [13]. It is not surprising, then, that 23% of female healthcare workers reported “caregiving exhaustion” compared to 14% of male healthcare workers [11]. 

In addition to challenges with balancing personal and family needs with work, female physicians face gender-based discrimination. Two-thirds of women in gynecology, a field dominated by women, experienced gender-based discrimination [18]. Women physicians earn 17% to 28% less than men, even after controlling for experience and part-time status [19]. They remain underrepresented in chair positions and other leadership roles across multiple specialties, including anesthesia, internal medicine, surgery, psychiatry, and pediatrics [20]. The pandemic clearly magnified these challenges; lack of flexibility, inadequate compensation, and lack of career advancement opportunities have remained consistent themes in studies examining the impact of the pandemic on female physicians [10]. These themes arose in our study as well, with respondents stating that they were not offered adequate compensation, flexibility, or opportunities for career advancement. 

Attrition from medicine has been understudied, but one study demonstrated a nearly 40% attrition rate of female physicians from academic medicine within the first five years of joining academia [21]. While only 8% of respondents in our study shifted to part-time or quit their jobs altogether, that number reflects a snapshot of a 23-month period. If the workforce continues to diminish at this rate, it will lead to an alarming gap in the number of physicians who work full-time. Even among those physicians who did not quit, the majority of our respondents thought about quitting their jobs at least monthly. Institutions cannot afford to ignore the data that preceded the pandemic or the trends that continued during the pandemic about gender discrimination in medicine, lack of work-life balance, and an overall lack of well-being among women physicians. Institutions should seek to rectify these issues in order to ensure a healthy, vibrant workforce.

Strengths of this study are the significant number of female physicians who responded to this survey, the representation of a variety of specialties, institutions, and geographic locales, and the depth and breadth of the questions that people responded to. We obtained a significant amount of information about the challenges of the pandemic for female physicians, especially for physicians with children, which had previously not been thoroughly studied in the literature. 

There are several limitations of this study. Because we recruited participants via listservs and social media, we do not know the extent to which the responses are representative of the population of female physicians in the US. Generalizability also may be limited by the fact that we limited our analysis to four specialties. Although our respondents were from across the nation, they were concentrated primarily in the Northeast. Geographic variations in state and institutional responses to the pandemic could impact our data. In addition, all the data collected were self-reported and reflected respondents’ experiences from a specific period of time. The study may not have captured the fluidity of experiences over time, but when compared to data preceding the pandemic, this study will likely remain relevant as it highlights the ongoing challenges for female physicians that originated prior to the pandemic.

Lastly, this study demonstrates several tangible changes that institutions can implement (such as offering free mental health services, backup childcare or childcare subsidies, and greater flexibility in scheduling) that may improve female physicians' quality of life. The guide to female physician retention (Figure 4, above) illustrates physician needs, with many solutions that institutions can implement readily. This study can serve as a call to action for institutions that seek to improve retention and physician experience. It can also serve as a springboard to explore other opportunities for change and create forward momentum to implement meaningful change. 

## Conclusions

We hope that our work underscores the ongoing importance of addressing barriers for female physicians, particularly those who have caretaking responsibilities. We encourage employers to offer flexibility, including options to work from home, increased compensation, decreased administrative burden, and backup childcare to decrease burnout and increase wellbeing and retention among female physicians. 

## Appendices

Survey questions for the pandemic and the female physician

Are you currently employed?

-Full time

-Part time

-Unemployed

Did your employment status change due to the pandemic?

-YES → went to full time/went to part time/became unemployed

-NO

Did the number of hours you worked change due to the pandemic?

-Increased

-Decreased

-Stayed the same

Did you incorporate telemedicine into your practice during the pandemic?

-YES

-NO

Do you have children? [if yes can go on to questions 6-11]

-YES→ How many? [write in] and Ages [write in]

-NO

At any point in the pandemic, did you decrease your work hours due to childcare responsibilities?

-YES [if yes, by how many hours?]

-NO

At any point in the pandemic, did you change your work hours due to childcare responsibilities?

-YES [can check multiple options]

--changed to working in the early morning

--changed to working in the evening

--changed to working overnight

--changed to telehealth

--changed to working weekends

--changed to per diem

--quit your job

--other [please specify]

-NO

At any point in the pandemic, was your childcare disrupted?

-YES →

--daycare closed

--nanny resigned

--family couldn’t provide childcare

--OTHER

-NO

At any point in the pandemic, was your child’s schooling disrupted?

-YES → school went to remote/school went to hybrid/school hours changed

-NO

Did your childcare options change during the pandemic?

-YES →

--changed to daycare

--changed to nanny

--changed to parent

--changed to other family member

-NO

Did your employer help you with childcare in any way during the pandemic?

-YES → [CAN SELECT MULTIPLE OPTIONS] provided access to backup daycare or nanny

--allowed you to work from home

--allowed you to change your hours to accommodate childcare responsibilities

--allowed you to split shifts with others or job share

--OTHER [please specify]

IF YES: which of the above did you take advantage of?

-No

At any point in the pandemic, did your employer offer you the following free or discounted options:

-laundry services

-access to virtual workouts or exercise subscriptions

-babysitting or nanny services

-daycare

-adult care

-meal prep or meal delivery services

-grocery delivery services

-housekeeping services

-access to clothing

-subscription services (i.e., Amazon Prime, KiwiCo, Coscto, etc.)

-mental health services

-coaching services

-meditation apps

-OTHER [please specify]

At any point in the pandemic, did your employer eliminate or decrease your: [CAN SELECT MULTIPLE OPTIONS]

-bonus

-RVU earnings

-retirement benefits

-pension

-healthcare benefits

-other benefits

-professional funds/continuing medical education funds

-vacation days

-paid time off

-research time

-administrative time

Did your employer:

-decrease your total compensation

-ask you to work more without offering additional compensation

Does your employer offer you any retention benefits?

-YES → if so, what?

-NO

Did you change jobs during the pandemic?

-YES → if yes, WHY? [should we give people options like:)

--lack of flexibility

--long commute

--hours didn’t work for you anymore

--excess administrative burden

--lack of support from employer

--inadequate compensation

--inadequate vacation time

--inadequate benefits

--family obligations

--partner found a different job

--OTHER [please specify]

-What could your employer have done to retain you? [write in]

Did you move during the pandemic?

-YES → If yes, WHY?

-NO

Surveys examining feelings adapted from Maslach burnout inventory and employee perceptions during the pandemic (Tables 3-4). 

**Table 3:** Burnout survey examining feelings at any point in the pandemic; adapted from Maslach burnout inventory.

	Always	Most of the time	Sometimes	Rarely	Never
Worn out at the end of the workday					
Emotionally drained at the end of the workday					
Fatigued when I get up in the morning					
Burnt out because of my job					
That I am working too hard					
That I am at the end of my rope					
Less empathy towards my patients					
Less empathy towards my colleagues					
Less connected with my patients					
Less connected with my colleagues					

**Table 4:** Employee perceptions during the pandemic

During the pandemic...	Strongly Agree	Agree	Neutral	Disagree	Strongly Disagree
I felt supported by my employer					
My employer acknowledged my work					
My employer provided me flexibility in my work					
I felt that my employer expected me to work harder than before					
My professional career was negatively impacted					
I was worried about becoming infected with COVID					
I was worried about spreading COVID to family members					
My employer provided me adequate personal protective equipment					
I was stressed because of childcare responsibilities					
I was stressed because of family responsibilities					

At any point during the pandemic, have you thought about quitting your job?

-Daily

-A few times a week

-Weekly

-Once a month or a few times a month

-A few times a year

-NEVER

If YES, why?

--lack of flexibility

--long commute

--hours didn’t work for me anymore

--excess administrative burden

--lack of support from employer

--inadequate compensation

--fear of COVID infection

--OTHER [please specify]

If you did leave your job, can you share why? → then go down to Q22

What could your employer have done, if anything, to retain you?

If you stayed at your job during the pandemic, can you share why? → then go down to Q24

What did your employer do, if anything, to retain you?

​​Were there any positive changes that occurred due to the pandemic?

DEMOGRAPHIC QUESTIONS

What is your age?

What state do you live in? [drop down]

Do you live in an urban, suburban, or rural setting?

Do you work in an urban, suburban, or rural setting?

Do you work for a:

-small group private practice (<6 physicians)

-large group private practice (6+ physicians)

-academic institution

-community institution

-state government institution

-federal government institution

What is your specialty? [drop down]

-Anesthesiology

-Dermatology

-Emergency Medicine

-Family Medicine

-Surgery

-Internal Medicine

-Neurology

-Obstetrics and Gynecology

-Ophthalmology

-Pathology

-Pediatrics

-Physical Medicine and Rehabilitation

-Psychiatry

-Urology

Do you have a subspecialty? YES/NO. If Yes, what is it?

Are you in training? YES/NO.

--If Yes, what year [drop down PGY 1-10]? 

--If NO, next question will be how many years have you been in practice since completing your training?

1-3

4-6

7-9

10-14

15-20

21+

How many hours a week do you work on average? [WRITE IN]

What is your race/ethnicity?

Black/African American

Hispanic/Latinx

White/European

South Asian

East Asian

Southeast Asian

Middle Eastern/North African

American Indian or Alaska  Native

Native Hawaiian/Pacific Islander

Multiracial

-Other [write in]

What is your marital status?

-Single

-Married or in a domestic partnership→  if yes go to questions 25-28

-Divorced

-Widowed

Are you in a same-sex relationship?

-YES/NO

Is your partner employed outside of the home?

-YES/NO

Did your partner’s employment status change during the pandemic?

-YES → became employed full-time/became employed part-time/became unemployed

-NO

Is your partner in medicine?

-YES/NO

Do you have any other dependents aside from children?

            -YES

            -NO

            -not formally, but I help to support family members

Would you be interested in participating in a focus group? If so, please include your email address and a staff member will reach out to you.
